# Surface Topography Has Less Influence on Peri-Implantitis than Patient Factors: A Comparative Clinical Study of Two Dental Implant Systems

**DOI:** 10.1021/acsbiomaterials.3c01809

**Published:** 2024-06-25

**Authors:** Badra Hussain, Jostein Ivar Grytten, Gunnar Rongen, Mariano Sanz, Håvard Jostein Haugen

**Affiliations:** † Department of Biomaterials, Institute of Clinical Dentistry, University of Oslo, Oslo 0316, Norway; ‡ Institute of Community Dentistry, University of Oslo, Oslo 0316, Norway; § Section of Periodontology, Faculty of Odontology, University Complutense of Madrid, Madrid 28040, Spain; ∥ ETEP (Etiology and Therapy of Periodiontal and Peri-Implant Diseases) Research Group, Complutense University, Madrid 28040, Spain

**Keywords:** dental implants, biofilm, peri-implantitis

## Abstract

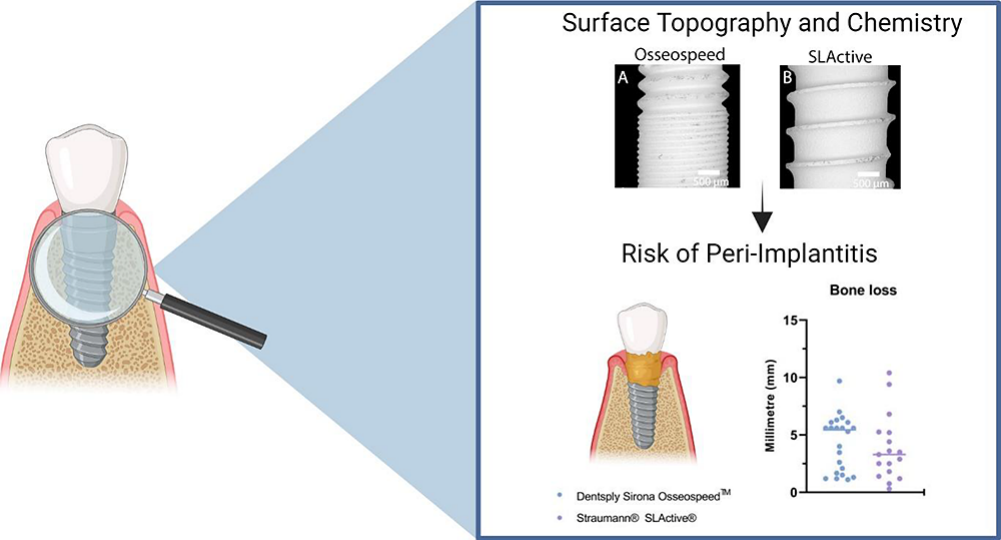

Objectives: This study aims to assess the risk of peri-implantitis (PI) onset among different implant systems and evaluate the severity of the disease from a population of patients treated in a university clinic. Furthermore, this study intends to thoroughly examine the surface properties of the implant systems that have been identified and investigated. Material and methods: Data from a total of six hundred and 14 patients were extracted from the Institute of Clinical Dentistry, Dental Faculty, University of Oslo. Subject- and implant-based variables were collected, including the type of implant, date of implant installation, medical records, recall appointments up to 2022, periodontal measurements, information on diabetes, smoking status, sex, and age. The outcome of interest was the diagnosis of PI, defined as the occurrence of bleeding on probing (BoP), peri-implant probing depth (PD) ≥ 5 mm, and bone loss (BL). Data were analyzed using multivariate linear and logistic regression. Scanning electron microscopy, light laser profilometer, and X-ray photoelectron spectroscopy were utilized for surface and chemical analyses. Results: Among the patients evaluated, 6.8% were diagnosed with PI. A comparison was made between two different implant systems: Dentsply Sirona, OsseospeedTM and Straumann SLActive, with mean follow-up times of 3.84 years (SE: 0.15) and 3.34 years (SE: 0.15), respectively. The surfaces have different topographies and surface chemistry. However, no significant association was found between PI and implant surface/system, including no difference in the onset or severity of the disease. Nonetheless, plaque control was associated with an increased risk of developing PI, along with the gender of the patient. Furthermore, patients suffering from PI exhibited increased BL in the anterior region. Conclusion: No differences were observed among the evaluated implant systems, although the surfaces have different topography and chemistry. Factors that affected the risk of developing PI were plaque index and male gender. The severity of BL in patients with PI was more pronounced in the anterior region. Consequently, our findings show that success in implantology is less contingent on selecting implant systems and more on a better understanding of patient-specific risk factors, as well as on implementing biomaterials that can more effectively debride dental implants.

## Introduction

Caries and periodontal disease represent the primary causes of tooth loss,^1^ linked to a decreased quality of life.^2^ Dental implants, designed to replace missing teeth, are critical in transferring loads to the jawbone while maintaining uniform force distribution within the surrounding bone tissue. Key requirements for these implants include high mechanical strength, fracture toughness, and biocompatibility. As a result, titanium (Ti) and its alloys have become the primary materials for dental implants owing to their exceptional biocompatibility and mechanical, physical, and chemical properties.^3,4^ When placed in the bone, titanium stimulates the formation of a bone-like apatite layer, enhancing bone-to-implant contact.^5^ Multiple factors influence the bone tissue response to dental implants. Research has highlighted the significance of surface chemistry and morphology in shaping the biological response.^6,7^ Enhancing surface roughness has emerged as a strategy to improve bone healing, accomplished through mechanical, chemical, or combined methods.^8−13^ Dental implant surfaces and designs are frequently modified to achieve optimal osseointegration,^7,14,15^ and techniques such as blasting and etching have yielded positive outcomes, optimizing surface roughness for enhanced bone response.^16−20^ Studies have consistently demonstrated that rough implant surfaces promote superior osseointegration and biomechanical fixation compared to smooth surfaces. An optimal surface roughness (*S*_a_) in the 1–4 μm range is believed to establish a stable interface between mineralized bone and the implant surface.^7,15,18,19,21−23^ After successful osseointegration, peri-implantitis (PI) emerges as a primary concern for dental implants,^24,25^ presenting as inflammation in the surrounding supporting tissue and subsequent loss of supporting bone.^26,27^ PI represents a pathological state observed in the vicinity of dental implants, marked by inflammation within the connective tissue surrounding the implant and a gradual reduction in the underlying bone support.^28^ When established, PI progresses rapidly.^29^ The significance of surface characteristics becomes even more pronounced when the implant is exposed to the oral cavity,^30,31^ as maintaining a clean surface free from biofilm formation becomes crucial in preventing the onset and progression of peri-implant diseases.^26^ Other patient-related factors also play a crucial role; plaque control, history of periodontitis, smoking, host immune system, and age have all been shown to affect the development and severity of PI.^31−35^ This multifactorial nature underscores the complexity of the disease, with the variety in implant surface characteristics and the challenge of effective surface cleaning,^36−38^ adding another layer of intricacy to the disease dynamics.

Biofilm accumulation is considered the main etiological factor for the development and progression of PI.^26,39^ These biofilms, which consist of bacterial communities embedded within a matrix of extracellular polymeric substances, can develop on various surfaces within the mouth, including dental implants.^40^ Dental implants are unique compared to orthopedic implants in that they are exposed to the diverse microbial environment of the oral cavity. The process of biofilm formation is initiated by the creation of a pellicle layer on the surface, acting as a base for bacterial adherence and the following maturation of the biofilm.^41,42^ The binding of proteins to this surface plays a key role in enabling bacterial attachment, thereby fostering the growth and development of the biofilm.^42^ This adherence creates a microenvironment that can harbor pathogenic microorganisms, leading to the chronic inflammation characteristic of PI. Laboratory studies show differences in biofilm development on different implant surfaces,^43,44^ implying a potential variance in PI development and progression between implant systems/surfaces. Some clinical studies indicate findings that align with these observations; for instance, a study comparing moderately rough implants to minimally rough implants implied more favorable outcomes in patients with a history of periodontitis when minimally rough implant surfaces were used.^45^ In addition, a recent study found differences in bone-level changes between implant systems,^46^ with favorable outcomes for Osseospeed implants. Other studies have failed to find such differences.^47,48^ Nonetheless, it is essential to note that a recent systematic review concluded that existing clinical studies cannot definitively establish a difference in PI incidence among different types of implant surfaces, with few studies assessing the impact of implant surfaces/systems on PI.^49^ This highlights the need for further research to shed more light on this topic.

This gives further reason to explore whether there is an increased risk of PI associated with specific implant systems. The main objective of this study was to evaluate whether there is a difference in the development and severity of PI among different implant systems installed at the Institute of Clinical Dentistry, Dental Faculty, University of Oslo. Additionally, the study aims to analyze the surface characteristics of the implant systems identified and analyzed in this study.

## Materials and Methods

### Data Collection and Participants

The research project received ethical approval from the Regional Committees for Medical and Health Research Ethics (reference number 2021/278092) and the Norwegian Centre for Research Data (2021/430060). The study was conducted in accordance with the principles outlined in the Helsinki Declaration, and STROBE guidelines were followed.

Data were obtained from the patient record system at the Institute of Clinical Dentistry, Dental Faculty, University of Oslo (SALUD, Titanium Oral Health Solutions, Dublin, Ireland). The data set was comprised of individuals who received dental implants at the faculty, starting with the implementation of the digital record system SALUD at the University of Oslo in 2009. Data from 2009 to 2022 were collected. Specifically, data were extracted from patients with implants installed at the faculty between 2009 and 2020. The collected data included the following variables: date of implant installation, implant type, medical records, recall appointments up to 2022, periodontal measurements available from the recall-appointment, information on diabetes and smoking status, sex, and age.

### Study Design and Setting

The study followed a historical cohort design, retrospectively examining patients who received implants at the time of installation as the starting point. The diagnosis of peri-implantitis (PI) based on the presence of bleeding on probing (BoP), peri-implant probing depth (PD) ≥ 5 mm, and bone loss (BL) was the disease event. The data from the patients and implants were evaluated based on follow-up data, specifically focusing on the occurrence of PI.

### Surface Evaluation of Implants

The implant systems identified in the clinical study were evaluated through the collection of commercial implant. These were then assessed based on their surface topography and chemistry, as detailed below.

### Surface Topography

The implant surface morphology was investigated with backscatter tabletop SEM (TM-3030, Hitachi, Japan) at 15 keV with three different magnifications on flat coins provided by the implant manufacturer. Two commercial implant surfaces (*n* = 6) were analyzed using a light laser profilometer (PLμ NEOX, Sensofar-Tech S.L., Terrassa, Spain) according to previously described procedures.^13,50^ The following implant surface morphological parameters were assessed: surface roughness (*S*_a_), surface skewness (*S*_sk_), surface kurtosis (*S*_ku_), the core fluid retention index (*S*_ci_), surface area increment (*S*_dr_), root-mean-square height (*S*_q_), and the total height of the surface (*S*_t_).

### Contact Angle

Contact angle measurements were conducted using the sessile drop mode with Laplace–Young model fitting (OCA Plus 15, DataPhysics Instruments GmbH, Filderstadt, Germany) on flat coins provided by the implant manufacturer. The measurements were performed with distilled water at 20 °C. An average of five consecutive measurements, using a 3 μL drop at a rate of 1 μL/s, was obtained. Six implant surfaces from each type of implant were used, and the median value between them was calculated.

### Surface Chemistry

The XPS analysis was performed on three different implants of each type on an Axis UltraDLD XP spectrometer (Kratos Analytical Limited, Manchester, United Kingdom). The instrument resolution was 1.1 eV for the survey scans and 0.55 eV for the detail scans for the employed settings, determined by measuring the full width at half-maximum FWHM of the Ag 3d5/2 peak obtained on sputter-cleaned silver foil. The emission of the photoelectrons from the sample was 90° (normal to the sample surface), and the incidence angle of the X-rays was 33.3° (or 56.7° between the X-ray incidence direction and captured photoelectron emission direction). A hybrid lens mode was used with a slot aperture at 80 eV pass energy for the survey spectra. The survey scan was executed at between 0 and 1100 eV binding energy. A hybrid lens mode with a slot aperture was used for the detail spectra at a pass energy of 20 eV. Detail spectra were recorded for O 1s, C 1s, Ti 2p, and N 1s. The energy shift due to surface charging was below 1 eV based on the C 1 s peak position relative to the established BEs; therefore, the experiment was performed without charge compensation in CASAXPS.

### Statistical Analysis

The difference between groups for surface roughness and chemistry was evaluated with an unpaired student *t* test (Graphpad Prism v. 20, San Jose, USA).

Descriptive statistics are presented as numbers (*n*) with percentages (%) and means with standard deviations (SD).

The event of interest was the incidence of PI based on all the osseointegrated implants using strict diagnostic criteria (BoP, PD ≥ 5 mm, and BL). Separate analyses were conducted constructing a regression analysis for each individual variable used in the diagnosis (BoP, PD, and BL). Explanatory variables, with a primary focus on implant type, were included in the regression analysis. Analysis was conducted at the implant level, focusing on the most severely affected site.

A stepwise linear regression model was employed for continuous outcomes (PD and BL). Logistic regression analysis was conducted for categorical outcomes, such as PI (yes/no) and BoP, with odds ratios (OR) converted to coefficient values. For the variable PD, a group variable was created based on the severity of PD. All regression analysis results are reported as coefficient values. The most affected site was chosen when applicable. Additional tests for significance were conducted using the Student’s *t* test and the Kolmogorov–Smirnov test as controls.

If necessary, adjustments for confounding variables were evaluated and implemented to obtain the best-fitted model. A *p*-value <0.05 was considered statistically significant for all analyses.

The statistical analysis was performed using STATA (version 17; College Station, TX, USA), and graphical illustrations were created using Prism 10 (GraphPad Software, San Diego, CA, USA).

## Results

### Clinical Evaluation

In the time period 2009–2020, 3054 implants were installed in 1213 patients. A total of 576 patients (1550 implants) received Astra Tech, Osseospeed, Molndahl, Sweden (now Dentsply Sirona, Osseospeed, Molndahl, Sweden (DSO), 520 (1252 implants) of Straumann SLActive, Basel, Switzerland (STS), 113 (248 implants) of Nobel biocare, TiUnite, Gothenburg, Sweden (NBT) and 4 (8 implants) of Biomet 3i, Osseotite, Palm Beach, FL, USA (BIO). Information regarding bone-/tissue-level implants was not available in the data set extracted.

Implants controlled from 6 months to <9 years after installment were identified. Implants only registered with the visit connected to the implant procedure (surgery and crown) were excluded. Patients with follow-up from 6 months to <9 years were included, yielding 644 patients (AT: 399, ST: 215, and NBT: 63). The mean follow-up time was 3.67 (SE: 0.11) yrs. In our data set, AT implants had the most prolonged follow-up period (3.84 years (SE: 0.15), compared to 3.34 (SE:0.15) for STS and 3.51 (SE:0.21) for NBT). From the data extracted, 44 of the implants met the criteria of having PI (6.8%). Table 1 displays the demographic representation of these patients. A total of 11.4% of the patients with PI had an NBT implant, 38.6% had an STS, and 50.0% had a DSO implant. DSO implants had a mean BL of 4.32 mm (SD: 0.52), while STS and NBT had BLs of 3.84 mm (SD: 0.69) and 4.82 mm (SD: 0.82), respectively.

**Table 1:** Demographics Data of Patients with Peri-Implantitis^a^

	variable	DSO	STS	NBT
patients	male/female	15/7	11/6	0/5
	smoker	5	9	0
	diabetes	0	0	0
	age (mean)	62.2	62.5	75.2
tooth	anterior/premolar/molar	11/8/3	7/8/2	2/3/0
mean PD mm		5.86 (SD = 1.67)	6.23 (SD = 2.05)	5.4 (SD = 2.3)
*p*-value^b^		0.82		
mean boneloss mm		4.32 (SD:0.52)	3.84 (SD:0.69)	4.82 (SD:0.82)
*p*-value^b^		0.98		

a DSO: Dentsply Sirona, Osseospeed, STS: Straumann SLActive, NBT: Nobel biocare, TiUnite.

b Adjusted *p*-value from multivariate regression analysis.

Due to the relatively low number of patients treated with NBT and BIO implants, these groups were excluded from the statistical analysis. Consequently, the comparison focused solely on DSO and STS, as these two groups yielded a sufficient and representative sample size for meaningful analysis. No significant difference was seen between the implant type and the development of PI. Tables 2 and 3 display regression analyses on patients with implants included in the analysis (*n* = 614), with BoP and PD as dependent variables. No difference between types of implants was observed in the risk of PD increase or BoP. However, male patients and implants installed in the molar region had a higher risk of deeper PD. The presence of plaque also affected the probability of having deeper PD and BoP. The regio variable was no longer significant when this was adjusted for in the PD regression model. The final adjusted coefficient is reported in Tables 2 and 3.

**Table 2:** Regression Analysis of Patients with Follow-Up after Implant Installment (*n* = 614) of Implant Type Dentsply Sirona, OsseospeedTM (DSO) and Straumann® SLActive (STS). The dependent variable is bleeding on probing (BoP).^a^

		*N*	% (of total)	logit coefficient (SE)	*p*- value	95% CI
implant type	DSO	399	64	0.31 (0.26)	0.23	[−0.20 to 1.81]
	STS	215	35			
plaque score	yes	77	13	2.05 (0.37)	0.01^b^	[1.32–2.78]
smoker	yes	155	25	0.18 (0.43)	0.68	[−0.66 to 1.02]
age	mean: 62.6			0.01 (0.02)	0.58	[−0.03 to 0.06]
sex	male (value: 1)	248	40	0.14 (0.27)	0.52	[−0.39 to 0.67]
	female(value:0)	261	43			
aegio	anterior	220	46	0.73 (0.58)	0.20	[−0.39 to 1.87]
	premolar	270	41	0.62 (0.57)	0.28	[−0.50 to 1.74]
	molar	124	13			
adjusted value for implant type coefficient:				0.25^c^ (0.27)	0.34	[−0.27 to 0.77]

a Regression coefficients with standard error in brackets, *p-*value, and confidence interval are reported. Final adjusted coefficient for implant type is reported.

b Significant *p*-value (<0.05).

c Adjusted for plaquescore.

**Table 3:** Regression Analysis of Patients with Follow-Up after Implant Installment (*n* = 614) of Implant Type Dentsply Sirona, OsseospeedTM (DSO) and Straumann® SLActive (STS). The dependent variable is probing depth (PD).^a^

		*N*	% (of total)	regression coefficient (SE)	*p*-value	95% CI
implant type	DSO	399	64	–0.02 (0.13)	0.86	[−0.28 to 0.24]
	STS	215	35			
plaque score	yes	77	13	3.10 (0.25)	0.01^b^	[2.62–3.59]
smoker	yes	155	25	–0.03 (0.21)	0.89	[−0.44 to 0.38]
age	mean: 62.6			–0.01 (0.01)	0.93	[−0.03 to 0.02]
sex	male (value: 1)	248	40	0.39 (0.12)	0.01^b^	[0.16–0.63]
	female(value:0)	261	43			
regio	anterior	220	46	0.43 (0.23)	0.07	[−0.03 to 0.88]
	premolar	270	41	–0.15 (0.22)	0.50	[−0.60 to 0.29]
	molar	124	13			
adjusted value for implant-type coefficient:				–0.03^c^ (0.12)	0.78	[−0.27 to 0.20]

a Regression coefficients with standard error in brackets, *p-*value, and confidence interval are reported. Final adjusted coefficient for implant type is reported.

b Significant *p*-value (<0.05).

c Adjusted for plaquescore and sex.

**Figure 1:**
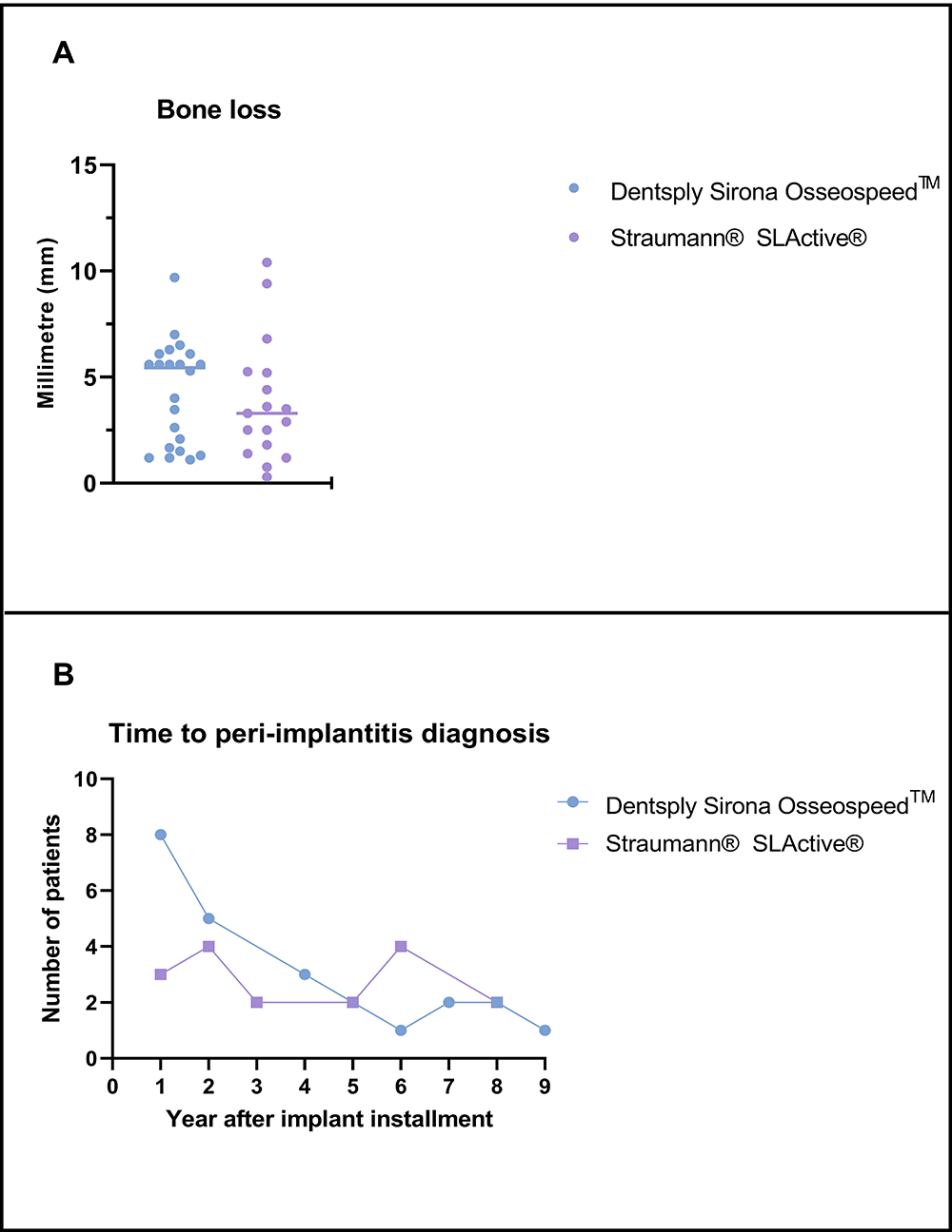
Forest plot of bone loss among different implant types in patients diagnosed with peri-implantitis (PI) (A). Time to PI diagnosis based on the type of implant used (B).

Table 4 displays the regression analysis of BL severity in patients with BoP and PD ≥ 5 mm. The severity of BL is the dependent variable. The regression coefficient for the severity of BL was close to zero, indicating no difference between implant types regarding BL (Figure 1A). The regio of placement (anterior region) significantly affects the probability of BL. The final implant-type coefficient is adjusted according to this.

**Table 4:** Regression Analysis of Patients with Peri-Implantitis, All Patients Included Have BoP and PD ≥ 5 mm, Dependent Variable Is Severity of Boneloss^a^^,^^b^

		*N*	% (of total)	regression coefficient (SE)	*p*-value	95% CI
implant type	DSO	22	56	–0.01 (0.56)	0.98	[−1.12 to 1.14]
	STS	17	44			
smoker	yes	14	35	–1.27 (0.81)	0.13	[−2.90 to 0.37]
age	mean: 63.7			0.07 (0.04)	0.07	[−0.00 to 0.14]
sex	male (value: 1)	26	67	–0.56 (0.85)	0.52	[−2.29 to 1.17]
	female(value:0)	13	33			
regio	anterior	18	46	3.08 (1.37)	0.03^c^	[0.27–5.91]
	premolar	16	41	1.48 (1.38)	0.29	[−1.35 to 4.30]
	molar	5	13			
adjusted value for implant type coefficient:				–0.14^d^ (0.63)	0.83	[−1.43 to 1.15]

a Regression coefficients follows after unadjusted coefficient for implant type with standard error in brackets, *p-*value, and confidence interval. Final adjusted coefficient for implant type is reported.

b DSO: Dentsply Sirona, Osseospeed, STS: Straumann SLActive.

c Significant *p*-value (<0.05).

d Adjusted for regio.

Table 5 displays the regression analysis with the severity of PD (patients with BoP and BL were included) as the dependent variable. The regression coefficient is close to zero, indicating no difference between implant types regarding PD. None of the confounding variables displayed significant coefficient variables.

**Table 5:** Regression Analysis of Patients with Peri-Implantitis, All Patients Included Have BoP and Bone Loss, Dependent Variable Is Severity of Peri-Implant Probing Depth^a^^,^^b^

		*N*	% (of total)	regression coefficient (SE)	*p*- value	95% CI
implant type	DSO	22	56	0.11 (0.46)	0.82	[−0.83 to 1.04]
	STS	17	44			
smoker	yes	14	35	0.71 (0.68)	0.30	[−0.66 to 2.07]
age	mean: 63.7			0.01 (0.03)	0.88	[−0.06 to 0.06]
sex	male (value: 1)	26	67	–0.52 (0.70)	0.47	[−1.94 to 0.89]
	female(value:0)	13	33			
regio	anterior	18	46	0.52 (1.04)	0.62	[−0.93 to 0.88]
	premolar	16	41	–0.51 (1.06)	0.63	[−0.93 to 0.88]
	molar	5	13			

a Regression coefficients with standard error in brackets, *p-*value, and confidence interval. None of the confounding variables displayed significant coefficient variables.

b DSO: Dentsply Sirona, Osseospeed, STS: Straumann SLActive.

Thirty-six percent of DSO implants and 18% of STS implants were diagnosed with PI 1 year after implant installment, and after 2 years, it increased to 59 and 41%, respectively (Figure 1B). However, there were no significant differences between the groups’ time to development of PI.

### Surface Characterization

**Figure 2:**
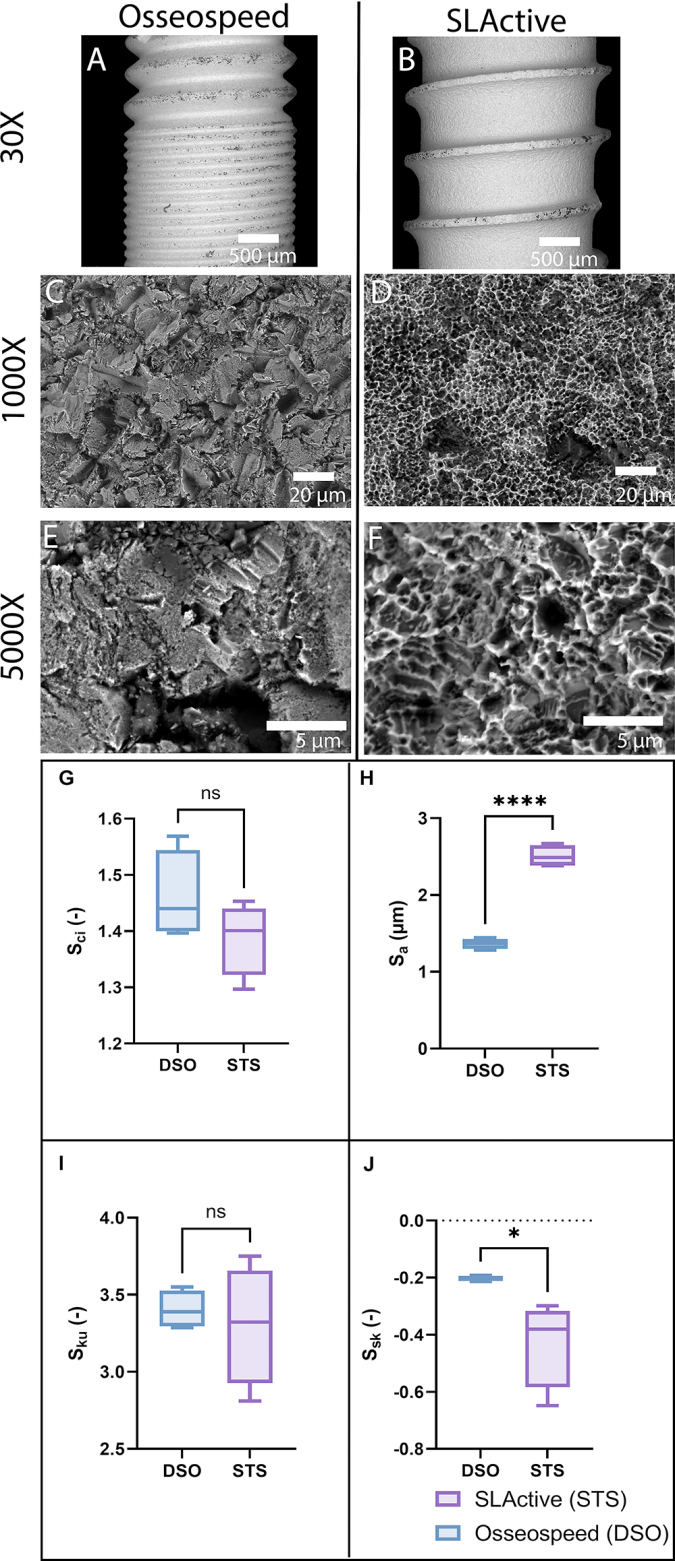
SEM image of Dentsply Sirona, Osseospeed (DSO) (A, C, E) and Straumann SLActive (STS) (B, D, F) at different magnifications. Boxplot of surface topography parameters of DSO and STS implants: G: *S*_a_ = surface roughness, H: *S*_sk_ = surface skewness, I: *S*_ku_ = surface kurtosis, and J: *S*_ci_ = core fluid retention index (*n* = 6, *p < 0.05, ****p < 0.001).

**Figure 3:**
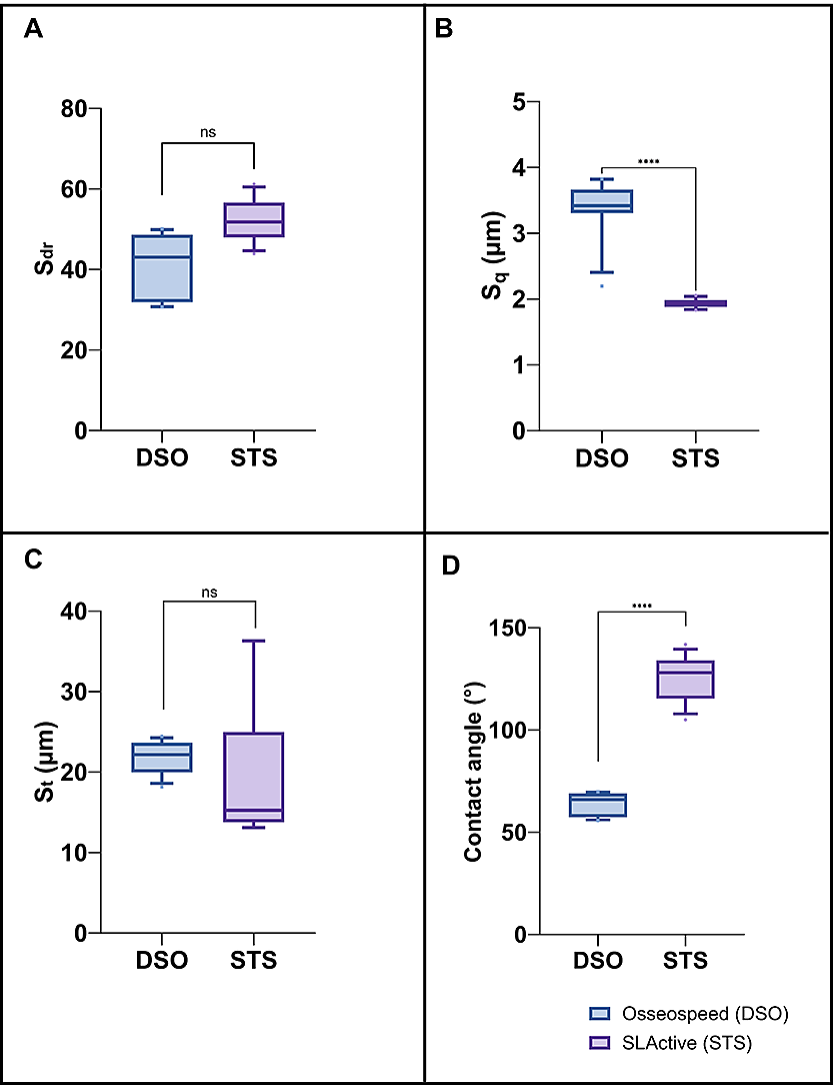
Boxplot of surface topography parameters of Dentsply Sirona, Osseospeed and Straumann SLActive implants: (A) *S*_dr_ = Surface area increment, (B) *S*_q_ = root-mean-square height, and (C) *S*_t_ = the total height of the surface and contact angle (*n* = 6, ****p < 0.001).

The surface morphology at different magnifications was visualized by SEM (Figure 2A–F), where Figure 2A,B gives an overview of the different threads of the two implants and Figure 2C–F shows the detailed structure of the implant surfaces. Figure 2 illustrates the surface parameters for the two implant surfaces being evaluated. In evaluating the surface characteristics of DSO and STS dental implants, the table’s data reflect surface roughness parameters at the 25th percentile, median, and 75th percentile. These parameters include the core fluid retention index (*S*_ci_), developed surface roughness (*S*_a_), Surface Kurtosis (*S*_ku_), and surface skewness (*S*_sk_). No significant difference was seen for the core fluid retention index (*S*_ci_) (Figure 2G). The STS implants showed a significantly higher surface roughness (*S*_a_) (2.385, IQR: 2.49–2.648) than DSO implants, meaning that STS implants have a consistently rougher surface than DSO implants (*p* < 0.0001). For Surface Kurtosis (*S*_ku_), which measures the asymmetry of the surface profile, no significant difference was seen (Figure 2I). The surface skewness (*S*_sk_) assesses the asymmetry of the surface deviation about the mean plane. The skewness is zero for a Gaussian surface with a symmetric shape of surface height distribution. *S*_sk_ showed that STS implants had significantly lower distributions (−0.3802, IQR: −0.5834, −0.3173). In contrast, DSO implants demonstrated median values of (−0.2026 IQR: −0.2113, −, −0.1939), proving a less peaked and less-tailed distribution than STS implants (*p* < 0.05) (Figure 2J). Regarding surface area increment (*S*_dr_) and total height of the surface (S_t_), there is no significant difference between the two implants, indicative of similar surface area due to texture and similar vertical distance between the highest and lowest point of the surface, respectively (Figure 3A,C). Figure 3B shows significantly higher root-mean-square height (*S*_q_) of DSO implants (3.37, SD:0.45) compared to STS implants (1.94, SD:0.10), indicative of more height deviation of the surface roughness from the mean line for DSO implants. DSO demonstrated a significantly lower contact angle (66°, IQR: 57.4–69.0) in comparison to STS (128°, IQR: 115.0–134), indicating a more pronounced hydrophilic surface characteristics (Figure 3D).

### Surface Chemistry

**Figure 4:**
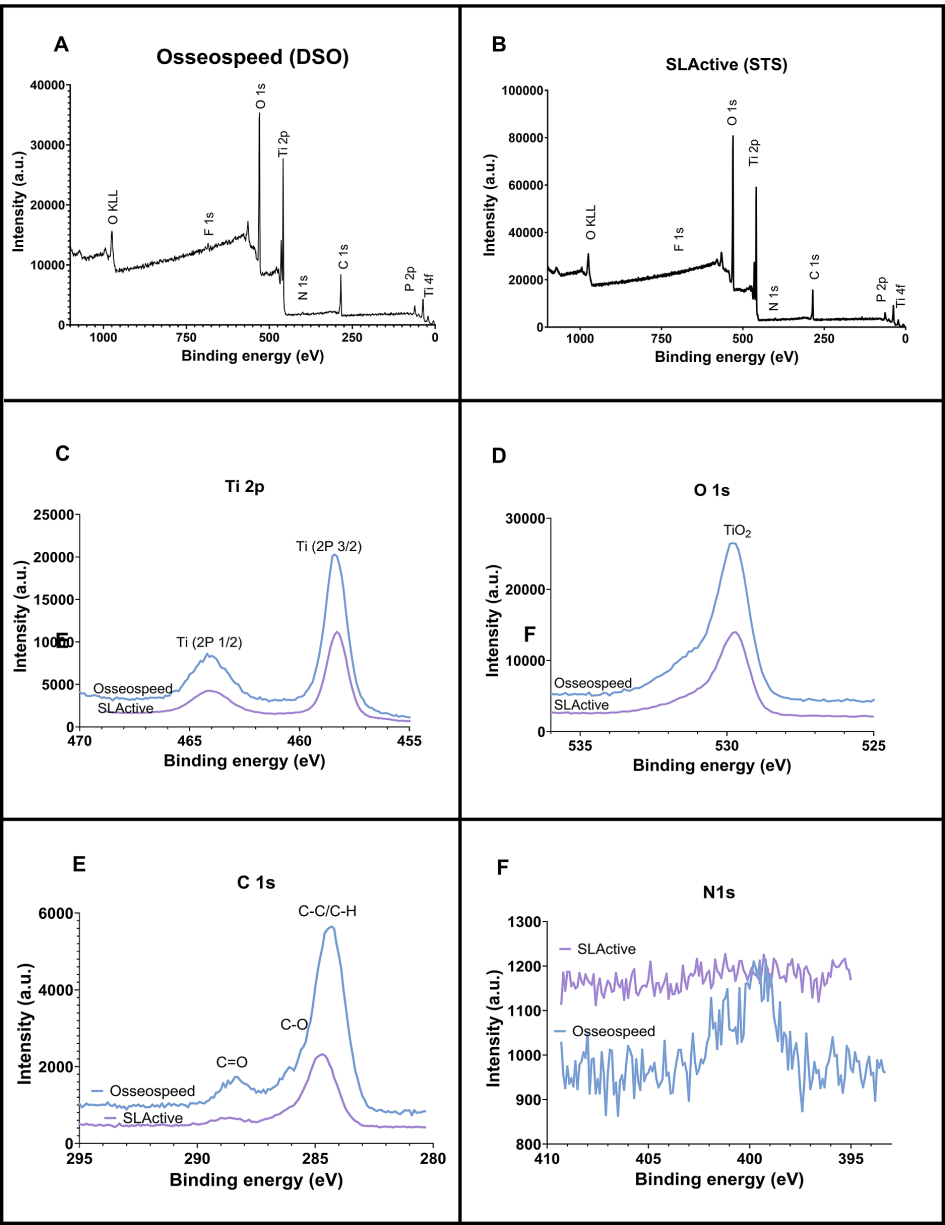
X-ray photoelectron spectroscopy survey scan of Dentsply Sirona, Osseospeed (A) and Straumann SLActive (B) and detailed scans for Ti 2p (C), O 1s (D), C 1s (E), and N 1s (F).

**Figure 5:**
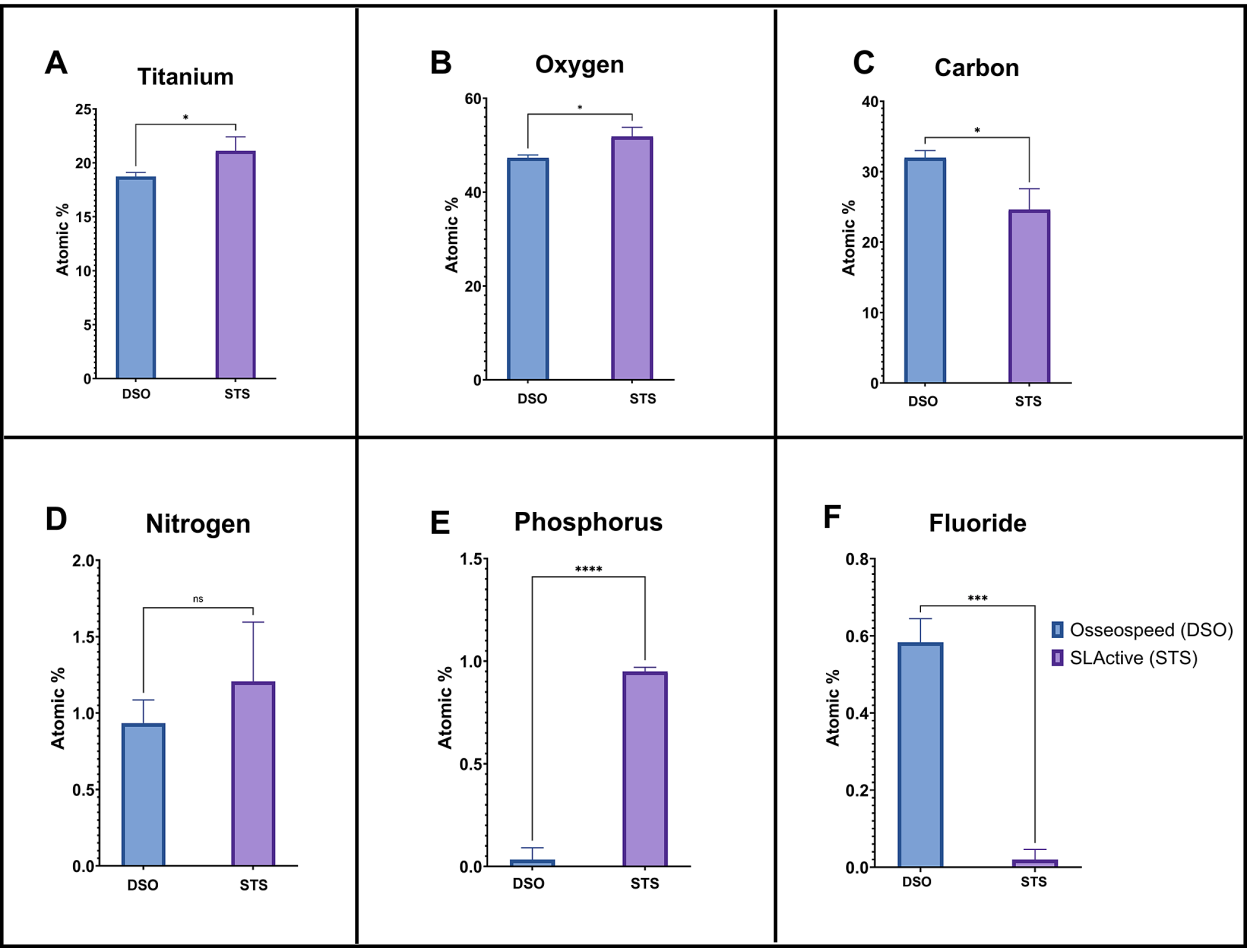
X-ray photoelectron spectroscopy elementary surface analysis of the implants showing mean atomic percentages with standard deviation of the following elements: titanium (A), oxygen (B), carbon (C), nitrogen (D), phosphorus (E), and fluoride (F) (*n* = 3, *p < 0.05, **p < 0.01, ***p < 0.001).

The XPS analysis revealed little difference in the bulk surface chemistry (Figure 4A–F) with more surface contaminants on the DSO surface (Figure 4 E). Titanium (Ti): STS implants have a significantly higher atomic percentage of titanium (20.1%) compared to DSO (18.7%) (*p* < 0.05), showing a more exposed titanium surface due to the surface treatment applied to STS implants (Figure 5A). The STS implants also have a higher atomic percentage of oxygen (51.9%) than DSO (47.%), providing a more prominent oxide layer on the STS implants (Figure 5B). There is a notable difference in the carbon content, with DSO implants having a higher atomic percentage (32.0%) than STS implants (24.6%) (*p* < 0.05) (Figure 5C), which correspond to higher organic contaminants or carbon-based residues on DSO implants (Figure 4E). There is no significant difference in nitrogen levels (Figure 3D); however, there is more phosphorus presence on STS implants (*p* < 0.0001) than on DSO. DSO implants show a small amount of fluoride (0.58%), whereas STS implants do not have any detectable fluoride.

## Discussion

The present study aimed to identify the onset of PI in patients treated with different implant systems with different surface morphology and evaluate the severity of PI when diagnosed. Overall, no significant differences in the development of PI among the implant systems were observed. The data set comprised four different implant systems: Osseospeed (DSO) (Dentsply Sirona, Mannheim, Germany), SLActive (STS) (Straumann Basel, Switzerland), TiUnite (NBT) (Nobel biocare, Gothenburg, Sweden), and Osseotite (BIO) (Biomet 3i, Palm Beach, FL, USA). Due to the low number of NBT and BIO samples, the comparison was only conducted between DSO and STS. Both DSO and STS implants are made of titanium, with somewhat different surface morphologies and chemical treatments. Investigating the surface morphology and treatment effects, it was evident that the blasting and acid etching techniques applied to DSO and STS implants modify their surface morphology. This results in unique etching pits or blasted facets, directly from the varied blasting and etching parameters. STS implants undergo blasting with alumina and subsequently acid etching, whereas DSO uses titania for blasting.^21^ The profilometer results collectively highlight distinct differences in the surface roughness profiles between DSO and STS dental implants (Figure 2), potentially impacting the biological responses and the osseointegration process.^51^ The data indicate that STS implants generally possess a rougher surface. Different roughness values for STS surfaces have been reported. Buser et al.^52^ found Ra = 3.1 μm on a solid screw implant, and Wieland et al.^53^ measured Ra = 4.33 ± 0.27 μm for similar treatments. Jarmer et al. showed that SEM investigations revealed dual levels of roughness, both macroscopic and microscopic.

Contact angle measurements suggest a more hydrophilic surface for DSO implants; however, this study’s findings reveal that clinically, this characteristic does not influence the development of PI between the two implant systems. This observation is noteworthy, especially considering that laboratory studies have previously demonstrated increased bacterial adhesion to hydrophobic implant surfaces.^54^

Several noteworthy differences emerge in examining the elemental composition of DSO and STS implants. A closer look at titanium content unveils that STS implants possess a slightly more significant titanium percentage of 21.11% compared to 18.73% in DSO implants. The increased titanium presence in STS may result from surface treatment processes that more effectively remove contaminants and uncover the underlying titanium. DSO implants exhibit a higher mean carbon content at 32%, in contrast to STS implants, which display a lower average of 24.6%. Similar results have been reported by Kang et al.^55^ This disparity may be linked to the varying presence of organic contaminants or the influence of distinct surface treatment methodologies on the adsorption of organic compounds.^56^ Conversely, DSO implants contain a reduced mean nitrogen content of 0.933% relative to the 1.207% found in STS implants, possibly reflecting the organic amines introduced during manufacturing or subsequent environmental adsorption.^57^ Jarmar et al. thoroughly analyzed the surface characteristics of DSO and STS dental implants using profilometry, scanning electron microscopy (SEM), and transmission electron microscopy (TEM).^58^ Ultrathin sections were prepared using focused ion beam (FIB) microscopy to obtain detailed microstructural and chemical information, which revealed notable differences in surface properties. The implant surfaces were found to be coated with crystalline TiO2 (including both anatase and rutile forms), amorphous titanium oxide, fluorine, and titanium hydride. The hydrofluoric acid alters the DSO microstructure and modifies the surface chemistry. A TEM sample prepared from an area revealed that the oxide layer can be extremely thin, down to 10 nm, and has an amorphous structure.^58^ Masaki et al.^59^ reported an atomic weight percentage of fluoride at 1% on the oxide surface. Ellingsen et al.^60^ indicated that surface fluoride promotes the precipitation of calcium and phosphorus, forming fluoridated hydroxyapatite and fluorapatite. TEM analysis revealed TiO2 islands composed of rutile and anatase with thicknesses between 0.5 and 1 μm. Eriksson et al.^61^ found that etching polished titanium with 10% HF for 3 min reduced oxide thickness from 36 to 29 nm, suggesting that the fluoride layer may be very thin or easily lost during preparation. TEM results indicated no surface oxide but revealed a defect-dense surface of unknown composition.^58^

When considering oxygen, STS implants reveal an elevated content, averaging 51.87%, as opposed to 47.33% in DSO implants. Such an increase could be attributed to differing surface treatments that impact the oxidation level or the prevalence of oxide layers and hydroxides.^62^ In fluoride content, DSO implants present a marginally higher percentage than an undetectable amount in STS implants; other studies also highlight the difference in contact angle between these two systems.^63−66^ The incorporation of fluorine is due to the hydrofluoric treatment reported in the literature.^22,67^ These differences in elemental composition suggest that the two types of implants, DSO and STS, undergo different surface treatments, resulting in slightly different surface chemistries that could influence their performance and osseointegration properties in clinical applications.^68,69^ It is important to note that the presence of elements such as carbon may not just indicate the elemental composition of the implant itself but also adsorbed or residual substances from the environment during handling of the implants during XPS analysis.^57,70^ Conforto et al.^71^ identified the SLA surface top layer as TiH_1.971_, though our electron diffraction studies could not confirm this. Similar findings have been seen by others.^72,73^ XPS analysis by others found the surface to be composed of TiO_2_ [7], with an oxide layer thickness of 4.5–5.5 nm, including TiO and Ti_2_O_3._^52^ Thermal desorption spectroscopy revealed significant hydrogen in the subsurface layer of SLA compared to polished surfaces.^74^ Numerous studies have demonstrated that surface chemistry and topography alterations impact protein adsorption, which in turn can influence immune response proteins.^75−77^ The most noteworthy difference between the two dental implants was the surface morphology. Both surfaces have shown respectable results regarding osseointegration.^22,78−80^

In vitro studies have revealed differences in bacterial colonization between different titanium surface modifications.^44,81^ Higher surface roughness facilitates biofilm formation,^44^ and nanoroughness also affects biofilm formation.^63^ In vitro models further indicate that surface modifications affect biological responses with different levels of titanium hydrides incorporated to the surface.^82^ DSO and STS physicochemical modifications can lead to variations in cellular behavior.^75,76^ Hotchkiss et al. reported that despite similar levels of cell attachment and DNA content after 7 days across all implants, differences in macrophage and MSC gene expression were evident.^66^ DSO increased proinflammatory gene expression and reduced anti-inflammatory gene expression, whereas STS had the opposite effect. These gene expression changes correlated with protein release patterns, suggesting that hydrophilic surfaces promote an anti-inflammatory phenotype conducive to faster healing and osseointegration.^83^ Surface wettability and oxide layer composition also influenced protein adsorption and subsequent cell responses.^84^ DSO, with higher oxygen content, did not increase wettability but promoted a proinflammatory environment, whereas STS favored an anti-inflammatory environment, according to Hotchkiss et al.^66^ This suggests that controlling inflammation through surface modifications can improve implant outcomes.^85^

Additionally, preclinical studies have shown differences in BL among different implant systems/surfaces,^86−88^ indicating that the variation in BL may be related to other surface characteristics or morphology rather than roughness as the sole reason.^88^ The clinical evidence has inclined more against this difference, as multiple studies have shown little difference among implant systems and the development/severity of PI.^89−91^ Our study’s findings align with these observations. However, clinical literature implying differences among systems is available;^45,46,92^ for example, Norton and Åström found significantly less BL for the DSO surface than STS and TiUnite surfaces.

In contrast, our study did not find such a difference. We observed that patients with PI did not exhibit any significant difference in BL among the two compared implant systems, DSO and STS (Tables 1 and 4, Figure 1A). This implies no apparent differences in the progression and severity of the disease based on the implant systems evaluated in this study. However, it is worth noting that the anterior region had more BL than the posterior regions (Table 4) in patients with PI, which is consistent with previous literature.^93,94^ This can be attributed to the bone morphology in anterior regions, where thin cortical bone is often combined with less dense trabecular bone,^95^ thus influencing the progress of BL once the disease is established. BL may also be influenced by other factors, such as bone quality and occlusal overload,^96^ which, combined with PI, can accelerate BL.^93^

BoP presence and increased PD were not different between the two implant types. However, we found a higher risk of increased PD in male patients and with implants installed in the molar region (Table 3). When adjusted for plaque score, the region was no longer significant. This indicates that cleaning difficulties in posterior regions impact development more in the posterior regions rather than solely the location. The presence of plaque affected the probability of having BoP and deeper PD. This underlines the importance of implant maintenance and oral hygiene instructions in accordance with previous literature associating plaque with disease development.^26,97^ The role of sex as a risk factor for developing PI has been documented.^98−100^ With contradictory findings, both males and females have been associated with the risk of development, while others have suggested no clear relationship.^101^ Our study had a prominent number of men developing PI. This implies an increased risk of developing PI in male patients. However, the severity of PD and BL was not influenced by the sex of the patient in patients diagnosed with PI. It did not affect the severity of the disease.

Another risk factor, smoking, has been associated with PI,^25^ while others have failed to detect this association.^31,100^ Studies also imply healthier peri-implant tissue in smoking patients.^33^ The present study found a higher percentage of smokers in the PI group (35%) compared to the overall percentage (25%). However, we did not find any significant association between smoking and PI onset or severity of the disease. Age was not associated with the risk of PI development, in accordance with previous literature.^25^ Diabetes has been debated.^25,33^ Our study did not find any relationship between diabetes and PI.

A total of 6.8% of the implants evaluated were diagnosed with PI in the present study. However, due to the short follow-up time for a number of patients, this number might differ from the actual number. Even with the loss of patients during follow-up, it is essential to note that the disease is often diagnosed early,^29,32^ giving the numbers more validity. Thirty-six percent of DSO implants and 18% of STS implants were diagnosed with PI 1 year after implant installment; after 2 years, this was increased to 59 and 41%, respectively. This is in accordance with the current consensus regarding the early onset of peri-implant disease.^29,32,102^ Additionally, when evaluating the differences in PI onset, there were no differences between the systems evaluated (Figure 1B). Despite variations in surface topography and in addition to the previously mentioned chemical differences, there is no impact on clinical outcomes related to PI. Current research in implantology emphasizes developing surfaces that resist bacterial colonization.^103^ Nonetheless, our findings suggest that an implant’s surface does not significantly influence the progression of the disease. This raises questions about the efficacy of newer surfaces with different antibacterial strategies, particularly their ability to resist bacterial colonization following disease onset.

Due to the nature of a retrospective cohort, the accuracy of historical data can give potential bias in the data collection.^104^ For instance, over the years, there have been changes in procedures, and disease registration criteria may have influenced the recorded numbers and outcomes in this present study, in addition to the variety among therapists.^104^ Ideally, a prospective study would provide more reliable data for analysis. Nevertheless, conducting such studies with well-defined protocols is time-consuming and often involves a limited number of patients. Therefore, it is crucial to develop robust register-based data in dentistry, which is essential for conducting larger analyses, such as retrospective cohort studies, which can be a source of easy and inexpensive data collection.^104^ These register-based data enable researchers to track treatments over an extended period and obtain reliable real-world evidence.

This highlights one of the main limitations of this study: the data were extracted from a single specialist/teaching clinic, potentially limiting its generalizability. The patients included in the study received treatment at a university clinic, and research has indicated that these individuals differ from those seeking care at private practices in terms of care-seeking patterns and continuity of care.^105^ This limitation could be addressed by gathering data from multiple clinics or through a register-based registry, which is limited in the field of dentistry in Norway. Nevertheless, it is worth noting that retrospective data collected from clinical practices have been shown to provide valuable and relevant insights.^106,107^ Additionally, the retrospective nature of the cohort could introduce bias due to changes in procedures and disease registration criteria over time. Future research would benefit from multicenter, prospective studies that include more extensive and diverse patient populations.

The development and implementation of small data into larger pools of data sets, along with the combination of big data registers, offer considerable value.^107^ This requires thorough quality checking at every step. Regular training for therapists and users is essential to ensure uniformity in criteria adherence and proficiency in data handling. Moreover, the personnel responsible for data management should possess the necessary qualifications. Adopting journal systems that facilitate easy data extraction for registers is recommended, as data extraction was challenging with the system used in this study. Keeping a record of routine changes is crucial, given the frequent updates in information and literature, especially in emerging fields such as implantology and PI. The potential for future research lies in developing robust register-based data pools and enhancing real-world evidence gathering for evidence-based clinical decision making. As dental healthcare moves into a more data-driven paradigm, the infrastructure for data management will play a critical role in advancing scientific inquiry and the day-to-day management of patient care. This practice will provide valuable insights into real-world evidence on dental healthcare diseases/treatments and yield more reliable data. As a result, it can significantly impact the provision of treatment and the overall healthcare experience for our patients. Within the limitations of this study, no differences among implant systems were observed regarding the onset and severity of PI. An association between plaque and sex was found, in addition to more BL in the anterior region for PI patients. Overall, these findings align with previous literature where differences among implant systems are evaluated.^108^

## Conclusions

In conclusion, the present study investigated the relationships between various implant systems—specifically Osseospeed (DSO) and SLActive (STS)—and the onset and severity of PI. Despite the difference in surface morphology and surface chemistry, which laboratory studies suggest may indicate a difference in the risk of PI, our clinical data showed no significant variation in disease onset or progression based on the type of implant system used. Hygiene/plaque scores and gender influenced the risk of developing PI. Among those with PI, regional variations in BL severity were observed, with anterior regions exhibiting more pronounced BL.

It can be concluded that the implant system evaluated in this study did not affect the severity of PI development. These results underscore the importance of focusing on patient-specific factors and oral hygiene maintenance in managing and preventing this disease. Therefore, the key to prevent PI lies less in the choice of implant systems and more in the comprehensive understanding of patient-specific factors and developing new and effective biomaterials that can safely and effectively remove the biofilm.

## Abbreviations

PI: peri-implantitis
BoP: bleeding on probing
PD: peri-implant probing depth
BL: bone loss
SEM: scanning electron microscopy
XPS: X-ray photoelectron spectroscopy
DSO: Dentsply Sirona, OsseospeedTM
STS: Straumann SLActive
NBT: Nobel biocare, TiUnite
BIO: Biomet 3i, Osseotite
